# Impact of Fish Intake Frequency on Cardiovascular Disease-Specific Survival in Hemodialysis Patients

**DOI:** 10.31662/jmaj.2023-0135

**Published:** 2023-12-11

**Authors:** Tadasuke Ando, Tomochika Murakami, Sakura Fujiyama, Shin-ya Sejiyama, Kan Murakami, Daisuke Miki, Yoshitsugu Fujita, Naomichi Yamaguchi, Ryoichi Shirakami, Satoki Abe, Masahiro Todaka, Shuntaro Suzuki, Hiroyuki Fujinami, Mayuka Shinohara, Shinro Hata, Toru Inoue, Tadamasa Shibuya, Toshitaka Shin, Hiromitsu Mimata

**Affiliations:** 1 Department of Urology, Faculty of Medicine Graduate School of Medicine, Oita University, Yufu, Japan; 2 Hemodialysis Center, JCHO Nankai Medical Center, Saiki, Japan; 3 Faculty of Medicine Graduate School of Medicine, Oita University, Yufu, Japan

**Keywords:** fish intake frequency, cardiovascular disease, hemodialysis, cardiovascular disease-specific survival

## Abstract

**Introduction:**

Cardiovascular disease (CVD) is the leading cause of death in hemodialysis patients (HPs). As a food source, fish contains both CVD-preventive and CVD-promoting fatty acids; however, there is no consensus on fish consumption as a preventive measure for CVD in HPs. This single-center longitudinal cohort study aims to assess the impact of fish intake frequency (FIF) per week on CVD in Japanese HPs.

**Methods:**

Upon the initiation of the study, 148 HPs were evaluated to determine the FIF, and blood samples were analyzed. These patients were then monitored for 6 years.

The relationships between each FIF and blood sampling data, CVD-specific survival (CSS), and new CVD-free survival (nCFS) were statistically calculated using Kaplan-Meier survival curves.

**Results:**

During the observation period, 65 deaths were reported, 16 of which were attributed to CVD. Further, 53 patients developed new CVD onset, and no association was found between the FIF and blood sampling data. Based on the Kaplan-Meier survival curves, there was a significant difference in the CSS probability rates at 72 months between patients with an FIF of ≥4 (0.719, 95% confidence interval (CI): 0.530-0.842) and those with an FIF of ≤3 (0.930, 95% CI: 0.851-0.968) (p < 0.01). However, the nCFS probability at 72 months did not significantly differ between patients with an FIF of ≥4 and those with an FIF of ≤3. Multivariate Cox proportional hazards regression showed that an FIF of ≥4 (hazard ratio: 3.64, 95% CI: 1.22-10.9, p = 0.02) was an independent predictor of CSS, but not of nCFS.

**Conclusions:**

It was suggested that a higher FIF in HPs might be one of the risks for developing CVD with increased mortality.

## Introduction

Cardiovascular disease (CVD) is the leading cause of death in hemodialysis patients (HPs), accounting for approximately 40% of all mortality cases ^(1), (2), (3), (4), (5), (6), (7), (8), (9)^. Dietary therapy plays an important role in the primary and secondary prevention of CVD ^(10)^. Further, it can be performed by patients themselves for the management of early-stage chronic kidney disease (CKD) and is cost-effective from the viewpoint of medical economics ^(1), (2), (3), (4), (5), (6), (7), (8), (9), (10)^. Concerning this, healthcare professionals should actively provide dietary guidance to HPs to prevent the occurrence of CVD from the early stages of CKD.

Fish has a high content of high-quality protein and essential fatty acids, which makes it important in the diet of HPs ^(1), (2), (3), (4), (5), (6), (7), (8), (9)^. Docosahexaenoic acid (DHA) and eicosapentaenoic acid (EPA) are essential omega-3 fatty acids that are abundant in fish and can prevent CVD via their anti-inflammatory effects ^(1), (2), (3), (4), (5), (6), (7), (8), (9)^. Another essential omega-6 fatty acid abundant in fish is arachidonic acid (ARA); however, ARA promotes inflammation via the ARA cascade ^(7), (8)^. Therefore, a high intake of ARA may promote CVD ^(7), (8)^.

There is no consensus on fish intake as a strategy to prevent CVD, as there have been reports that show increased fish intake prevents CVD ^(11), (12)^ as well as reports that claim the opposite ^(11), (12)^. Furthermore, the evidence available for dietary studies is limited because it is extremely difficult to conduct double-blind, randomized controlled trials ^(11), (12)^. For these reasons, the American Heart Association’s recommendation for preventing the occurrence of CVD is limited to “healthy people should eat fish 2-3 days per week” ^(13)^.

It has been predicted that the aging society will further increase due to the declining birth rate ^(14)^, and dietary guidance that is easy for elderly HPs to understand and implement will be necessary. Simple dietary advice using food intake frequency is thought to be easy for elderly people to understand, enforce, and comply with.

However, the relationship between fish intake frequency (FIF) per week and CVD in HPs is currently unknown, and real-world data are lacking, especially in Japanese HPs.

The current study aims to determine whether there is an association between FIF and new CVD onset and mortality in HPs. Herein, we report the results of this single-center longitudinal 6-year cohort study on the association between FIF and overall survival (OS), CVD-specific survival (CSS), and new CVD-free survival (nCFS) in HPs.

## Materials and Methods

### Study design

This single-center longitudinal cohort study included all adult patients who underwent hemodialysis between January 2015 and December 2021 at Nankai Medical Center and aims to determine whether there is an association between FIF and new CVD onset and mortality in HPs. This study was conducted following the Declaration of Helsinki and good clinician guidelines. Further, the Ethics Committee of Nankai Medical Center approved this study (study number: NC-H27-001).

### Participants

We included HPs aged ≥20 years who underwent hemodialysis at Nankai Medical Center for at least 30 days and had stable conditions, could participate in the study, could provide written informed consent, and could answer the questionnaire in January 2015. We excluded patients with a history of kidney transplantation, with active infections or cancer, and those being treated for acute CVD from the study. Data on the characteristics of patients (age, sex, hemodialysis duration, medical history [including diabetes and CVD], and medications [phosphate binders and antihyperlipidemic drugs]) were collected from the medical chart. The FIF survey was conducted only once at the beginning of this study on the routine monthly blood collection day by asking the patients to indicate their average daily FIF (0-1 day, 2-3 days, 4-5 days, and almost every day) as previously reports for the general population or over middle age ^(15), (16), (17), (18), (19)^. Expert nurses, clinical engineers, and urologists guided HPs who needed help in completing the FIF survey. CVD was defined as the occurrence of heart failure, ischemic heart disease, valvular disease, large vessel disease, or stroke. Information on CVD history was obtained from the medical chart on the day of the FIF survey. A blood sample was collected immediately before hemodialysis and during fasting. White blood cell count, hemoglobin, C-reactive protein (CRP), albumin, total cholesterol, high-density lipoprotein cholesterol, low-density lipoprotein cholesterol, triglycerides, EPA, DHA, ARA, potassium (K), and phosphorus (P) levels were evaluated. After the FIF survey and blood sample collection, patients were followed up until December 2021 to assess for overall mortality, CVD-related mortality, and new CVD onset.

### Statistical analysis

Categorical variables were compared using Fisher’s exact test and the student’s t-test or the Mann-Whitney U test was used for continuous variables.

Logistic regression models were used to calculate adjusted odds ratios (ORs) with 95% confidence intervals (CIs) for the risk of overall mortality, CVD mortality, and new CVD onset in HPs. Multivariate logistic regression model analyses were performed to adjust for selecting an appropriate number of clinically important confounders, as shown in Table 1.

**Table 1.** Characteristics of the Patients in the Overall Survival Group and Overall Death Group.

Variable (unit: reference value)	Overall (n = 148)	Overall survival group (n = 83)	Overall death group (n = 65)	p-value
Male/Female	85/63	47/36	38/27	0.88
Age (years)	68.5 ± 11.4	64.4 ± 11.2	73.8 ± 9.6	<0.01
Body Mass Index (kg/m^2^)	24.7 ± 4.2	22.6 ± 3.2	24.0 ± 2.9	0.39
Duration on HD (months)	94.3 ± 96.3	84.6 ± 85.0	106.6 ± 109.8	0.17
Patients with Diabetes	54 (36.5%)	30 (36.1%)	24 (36.9%)	1
Patients with a history of CVD	78 (52.7%)	33 (39.8%)	45 (69.2%)	<0.01
Patients taking phosphate binders	96 (64.9%)	62 (74.7%)	34 (52.3%)	<0.01
Patients taking antihyperlipidemic drugs	8 (5.4%)	7 (8.4%)	1 (1.5%)	0.08
Patients taking antihypertensive drugs	63 (42.6%)	36 (43.4%)	27 (41.5%)	0.78
FIF (days/week)				<0.01
0-1 day	32 (21.6%)	26 (31.3%)	6 (9.2%)
2-3 days	68 (45.9%)	43 (51.8%)	25 (38.5%)
4-5 days	29 (19.6%)	9 (10.8%)	20 (30.8%)
everyday	19 (12.8%)	5 (6.0%)	14 (21.5%)
Blood sampling data
WBC (/μL: 3100-8400)	5056 ± 1642	5186 ± 1687	4821 ± 1672	0.34
Hemoglobin (g/dL: 11.4-16.6)	10.2 ± 1.62	10.38 ± 1.68	10.19 ± 1.26	0.64
CRP (mg/L: 0.0-0.3)	0.33 ± 0.58	0.22 ± 0.45	0.48 ± 0.74	<0.01
Albumin (g/L: 3.8-5.3)	3.5 ± 0.40	3.35 ± 0.69	3.50 ± 0.41	0.13
P (mg/L: 2.5-4.5)	5.7 ± 1.5	5.86 ± 1.57	5.42 ± 1.37	0.08
K (mEq/L: 3.5-5.0)	4.9 ± 0.8	4.99 ± 0.85	4.75 ± 0.74	0.07
TG (mg/dl: 20-149)	97.9 ± 60.8	106.9 ± 69.4	85.8 ± 447	0.03
T-Chol (mg/dl: 140-199)	143.1 ± 32.8	150.3 ± 33.8	134.5 ± 29.0	<0.01
HDL-c (mg/dl: ≥40)	41.8 ± 12.8	43.9 ± 13.6	39.8 ± 11.4	0.06
LDL-c (mg/dl: 60-119)	81.0 ± 25.6	85.1 ± 26.1	76.3 ± 24.1	0.04
DHA (μg/mL: 54.8-240.3)	92.5 ± 33.4	91.2 ± 36.2	93.7 ± 29.7	0.66
EPA (μg/mL: 10.2-142.3)	52.9 ± 32.4	52.4 ± 32.5	52.6 ± 32.4	0.97
ARA (μg/mL: 135.7-335.3)	144.4 ± 40.6	147.6 ± 41.6	139.0 ± 39.1	0.20

In addition, the Kaplan-Meier survival curves were generated to calculate the probabilities of overall survival (OS), CVD-specific survival (CSS), and new CVD-free survival (nCFS) based on each FIF, and statistically significant differences between the Kaplan-Meier survival curves were evaluated using the log-rank test. An appropriate number of clinically important confounders were selected, and the magnitude of their respective effects on each survival was assessed using COX proportional hazards regression. Fine-Gray proportional hazards regression analysis was used to evaluate the influence of competing risks such as death due to causes other than CVD for CSS and all-cause death for nCFS.

All statistical analyses were performed using EZR (Saitama Medical Center, Jichi Medical University), a GUI for R (The R Foundation) for Statistical Computing version 2.13.0.20). More precisely, it is an improved version of the R commander (version 1.6-3), equipped with statistical functions frequently used in biostatistics ^(20)^. The statistical significance level was set at p < 0.05.

## Results

This study included 85 men and 63 women with a mean age of 68.5 (standard deviation: 11.4) years and a mean hemodialysis duration of 94.3 (96.3) months. The median FIF was 2-3 days. In total, 35 (41.2%) men and 32 (50.8%) women had a history of CVD. During the 6-year observation period, 65 deaths (infectious disease: 28, cancer: 11, and others: 10) were recorded, 16 of which were due to CVD (heart failure: 6, ischemic heart disease: 4, valvular disease: 1, large vessel disease: 2, and stroke: 3). Moreover, 53 patients developed new CVD onset (heart failure: 26, ischemic heart disease: 19, valvular disease: 1, large vessel disease: 1, and stroke: 6). Compared with survivors, non-survivors were older, had a greater proportion of patients with a history of CVD and FIF, and a lower proportion of patients taking phosphate-binding agents (Table 1). The CVD mortality group had a significantly higher FIF than the non-CVD mortality group (Table 2). However, there were no significant differences between the new CVD onset group and non-new CVD onset groups (Table 3).

**Table 2.** Characteristics of Patients in the Non-CVD Mortality Group and CVD Mortality Group.

Variable (unit: reference value)	Non-CVD mortality group (n = 132)	CVD mortality group (n = 16)	p-value
Male/Female	77/55	8/8	0.60
Age (years)	68.2 ± 11.4	71.5 ± 11.7	0.27
Body Mass Index (kg/m^2^)	23.1 ± 2.6	22.7 ± 2.9	0.56
Duration on HD (months)	92.1 ± 91.4	112.4 ± 137.2	0.43
Patients with Diabetes	50 (37.9%)	4 (25.0%)	0.41
Patients with a history of CVD	66 (50.0%)	12 (75.0%)	0.07
Patients taking phosphate binders	88 (66.7%)	8 (50.0%)	0.27
Patients taking antihyperlipidemic drugs	8 (6.1%)	0 (0%)	0.6
Patients taking antihypertensive drugs	56 (42.4%)	7 (43.8%)	0.78
FIF (days/week)			<0.01
0-1 day	30 (22.7%)	2 (12.5%)
2-3 days	64 (48.5%)	4 (25.0%)
4-5 days	26 (19.7%)	3 (18.8%)
everyday	12 (9.1%)	7 (43.8%)
Clinical data
WBC (/μL: 3100-8400)	5071 ± 1574	5063 ± 2458	0.98
Hemoglobin (g/dL: 11.4-16.6)	10.32 ± 1.02	10.26 ± 1.22	0.59
CRP (mg/L: 0.3-1.7)	0.32 ± 0.60	0.51 ± 0.72	0.24
Albumin (g/L: 3.8-5.3)	3.27 ± 0.38	3.49 ± 0.29	0.44
P (mg/L: 2.5-4.5)	5.71 ± 1.54	5.29 ± 1.08	0.30
K (mEq/L: 3.5-5.0)	4.88 ± 0.83	4.88 ± 0.69	0.99
TG (mg/dl: 20-149)	99.3 ± 61.7	83.9 ± 49.7	0.34
T-Chol (mg/dl: 140-199)	144.1 ± 32.5	137.7 ± 34.6	0.46
HDL-c (mg/dl: ≥40)	42.0 ± 12.8	42.5 ± 13.4	0.89
LDL-c (mg/dl: 60-119)	81.9 ± 25.1	75.4 ± 29.3	0.33
DHA (μg/mL: 54.8-240.3)	92.1 ± 33.9	94.0 ± 30.2	0.83
EPA (μg/mL: 10.2-142.3)	52.6 ± 33.2	52.0 ± 24.3	0.94
ARA (μg/mL: 135.7-335.3)	144.8 ± 40.0	135.9 ± 45.7	0.41

**Table 3.** Characteristics of Patients in the Non-New CVD Onset Group and New CVD Onset Group.

Variable (unit: reference value)	Non-new CVD onset group (n = 95)	New CVD onset group (n = 53)	p-value
Male/Female	55/40	30/23	1.0
Age (years)	68.5 ± 12.4	68.7 ± 9.7	0.91
Body Mass Index (kg/m^2^)	22.9 ± 2.9	23.2 ± 3.1	0.44
Duration on HD (months)	107.7 ± 91.4	70.2 ± 76.9	0.02
Patients with Diabetes	33 (34.7%)	21 (39.6%)	0.60
Patients with a history of CVD	56 (58.9%)	22 (41.5%)	0.059
Patients taking phosphate binders	67 (70.5%)	29 (54.7%)	0.07
Patients taking antihyperlipidemic drugs	4 (4.2%)	4 (7.5%)	0.46
Patients taking antihypertensive drugs	40 (42.1%)	23 (43.4%)	0.67
FIF (days/week)			0.13
0-1 day	24 (25.3%)	8 (15.1%)
2-3 days	38 (40.0%)	30 (56.6%)
4-5 days	22 (23.2%)	7 (13.2%)
everyday	11 (11.6%)	8 (15.1%)
Clinical data
WBC (/μL: 3100-8400)	5235 ± 1523	4775 ± 1992	0.11
Hemoglobin (g/dL: 11.4-16.6)	10.46 ± 1.00	10.19 ± 1.23	0.60
CRP (mg/L: 0.3-1.7)	0.38 ± 0.66	0.26 ± 0.50	0.28
Albumin (g/L: 3.8-5.3)	3.48 ± 0.21	3.32 ± 0.27	0.36
P (mg/L: 2.5-4.5)	5.71 ± 1.61	5.58 ± 1.28	0.64
K (mEq/L: 3.5-5.0)	4.80 ± 0.74	5.03 ± 0.91	0.10
TG (mg/dl: 20-149)	98.1 ± 64.5	96.9 ± 53.2	0.91
T-Chol (mg/dl: 140-199)	143.2 ± 33.7	143.7 ± 30.9	0.93
HDL-c (mg/dl: ≥40)	41.3 ± 13.0	43.5 ± 12.3	0.32
LDL-c (mg/dl: 60-119)	82.5 ± 24.6	78.9 ± 27.2	0.42
DHA (μg/mL: 54.8-240.3)	88.3 ± 32.2	99.4 ± 34.7	0.053
EPA (μg/mL: 10.2-142.3)	50.4 ± 34.2	56.4 ± 28.5	0.28
ARA (μg/mL: 135.7-335.3)	143.1 ± 38.2	145.2 ± 44.9	0.77

Multivariate logistic regression analysis showed that CRP level, CVD history, and age were associated with a higher risk of overall death, with adjusted ORs of 3.3 (95% CI: 1.5-7.2, p < 0.01), 2.81 (95% CI: 1.3-6.3, p = 0.01), and 1.12 (95% CI: 1.1-1.2, p < 0.01), respectively.

Further, only FIF ≥ 4 was associated with a higher risk of CVD mortality, with adjusted ORs of 3.64 (95% CI: 1.22-10.9, p = 0.02) (Table 4). Further, only hemodialysis duration was associated with a reduced risk of new CVD onset, with adjusted ORs of 0.99 (95% CI: 0.989-0.998, p < 0.01). There was no association between FIF, age, and sex (data not shown).

**Table 4.** Single and Multiple Regression Analysis between the CSS and Clinical Laboratory Parameters.

	Single regression analysis		
Explanatory variable	Odds ratio	95% confidence interval	P value
Male/Female	0.71	0.25-2.02	0.53
Age (years)	1.03	0.98-1.08	0.27
Body Mass Index (kg/m^2^)	1.12	0.93-1.28	0.27
Duration on HD (months)	1.00	1.00-1.01	0.43
Patients with Diabetes	0.55	0.17-1.79	0.32
Patients with a history of CVD	3.00	0.92-9.78	0.07
Patients taking phosphate binders	0.50	0.17-1.42	0.19
Patients taking antihyperlipidemic drugs	<0.01	0.00-0.01	0.99
Patients taking antihypertensive drugs	0.69	0.19-2.21	0.56
FIF (days/week) ≥ 4	4.12	1.40-12.1	>0.01
WBC (/μL: 3100-8400)	1.00	1.00-1.00	0.98
Hemoglobin (g/dL: 11.4-16.6)	1.02	0.89-1.17	0.77
CRP (mg/L: 0.0-0.3)	1.46	0.76-2.80	0.26
Albumin (g/L: 3.8-5.3)	1.11	0.60-1.24	0.25
P (mg/L: 2.5-4.5)	0.81	0.55-1.20	0.29
K (mEq/L: 3.5-5.0)	1.02	0.51-2.04	0.97
TG (mg/dl: 20-149)	0.98	0.97-1.01	0.21
T-Chol (mg/dl: 140-199)	1.03	0.97-1.11	0.34
HDL-c (mg/dl: ≥40)	0.96	0.89-1.05	0.38
LDL-c (mg/dl: 60-119)	0.96	0.90-1.03	0.24
DHA (μg/mL: 54.8-240.3)	1.02	0.99-1.04	0.24
EPA (μg/mL: 10.2-142.3)	0.99	0.97-1.02	0.45
ARA (μg/mL: 135.7-335.3)	0.99	0.97-1.01	0.49
	Multiple regression analysis		
FIF ≥ 4	3.64	1.22-10.9	0.021
Patients with a history of CVD	2.49	0.744-8.36	0.14

**Figure 1.**
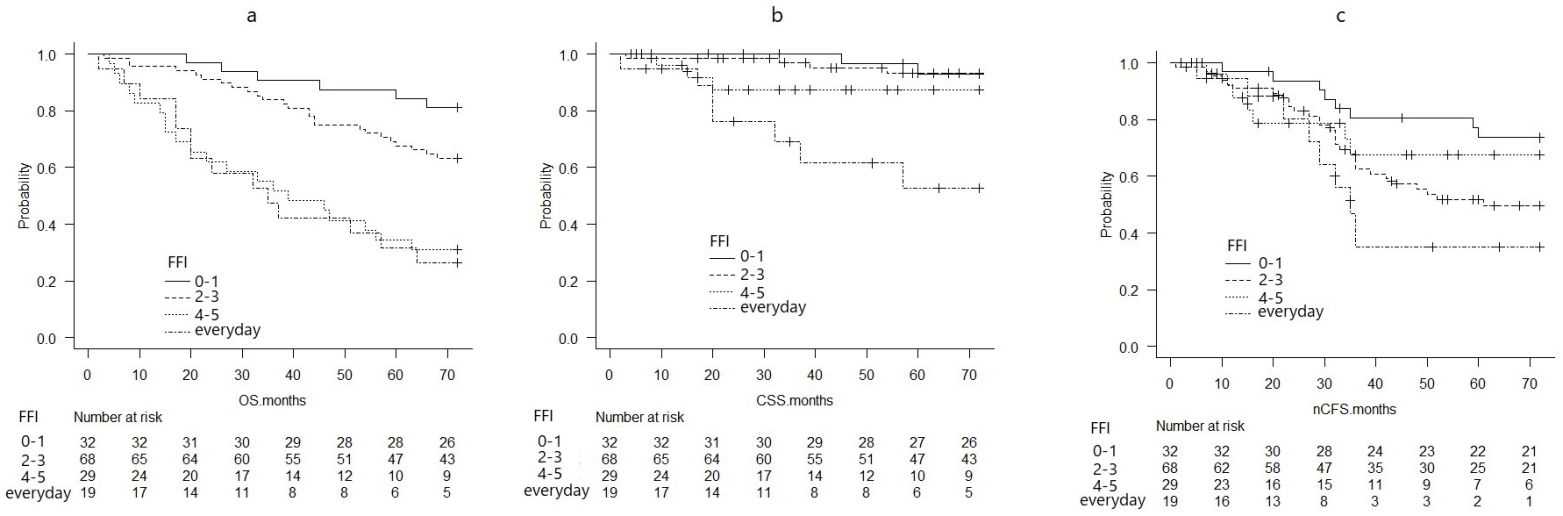
Kaplan-Meier survival curves per FIF (a: OS, b: CSS, c: nCFS) OS, CSS, and nCFS were different for each FIF.

**Figure 2.**
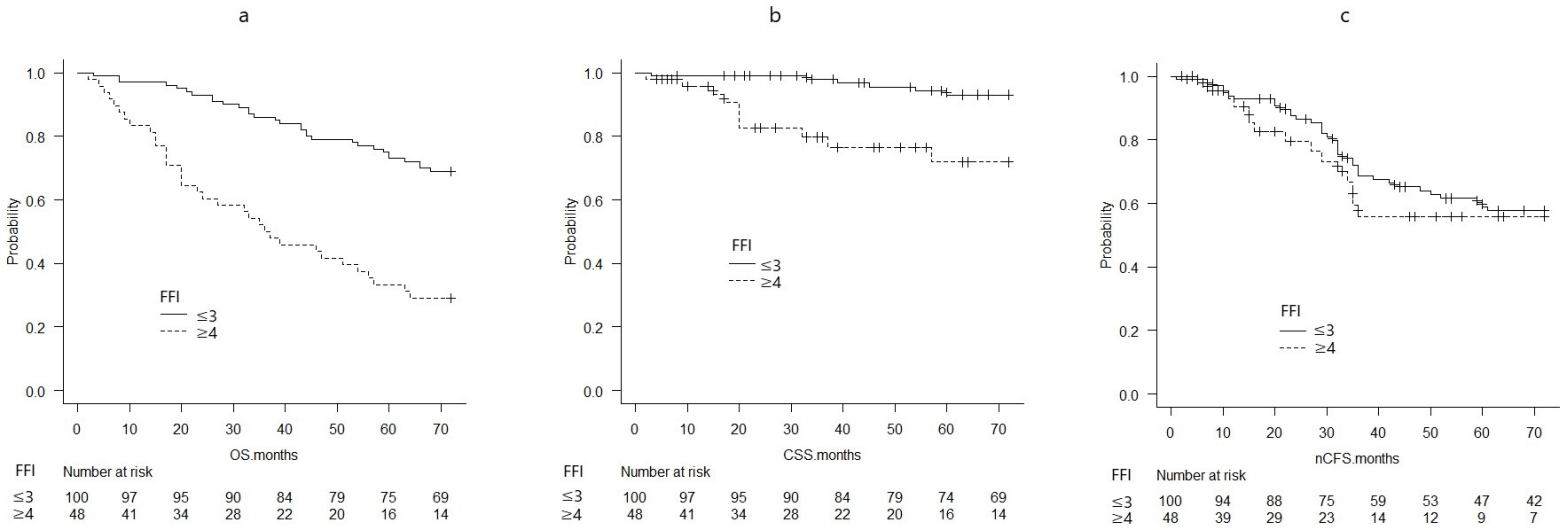
Kaplan-Meier survival curves compared between an FIF of ≤3 and an FIF of ≥4 (a: OS, b: CSS, c: nCFS) Both OS and CSS were significantly different between the two groups with p < 0.01 in the log-rank test. There was no significant difference in nCSF between the two groups in the log-rank test (p = 0.454).

The probability of OS, CSS, and nCFS per FIF was calculated using the Kaplan-Meier survival curves (Figure 1a, b, c). OS and CSS worsened with increasing FIF (Figure 1a, b, c). Based on the median FIF, the OS, CSS, and nCFS were compared between the group with an FFI of ≤3 and that with an FFI of ≥4 (Figure 2a, b, c).

The probabilities of OS at 72 months significantly differed between patients with an FIF of ≥4 (0.292, 95% CI: 0.172-0.423) and those with an FIF of ≤3 (0.690, 95% CI: 0.589-0.771) (p < 0.01) (Figure 2a).

The CSS probabilities at 72 months significantly differed between patients with an FIF of ≥4 (0.719, 95% CI: 0.530-0.842) and those with an FIF of ≤3 (0.930, 95% CI: 0.851-0.968) (p < 0.01) (Figure 2b).

The nCFS probabilities at 72 months did not differ between patients with an FIF of ≥4 (0.558, 95% CI: 0.370-0.710) and those with an FIF of ≤3 (0.577, 95% CI: 0.467-0.672) (p = 0.454) (Figure 2c).

Multivariate Cox proportional hazards regression analysis showed that an FIF of ≥4 (hazard ratio [HR]: 2.403, 95% CI: 1.430-4.038; p < 0.01), CRP level (HR: 1.833, 95% CI: 1.291-2.602; p < 0.01), age (HR: 1.076, 95% CI: 1.046-1.106; p < 0.01), and hemodialysis duration (HR: 1.004, 95% CI: 1.002-1.007; p < 0.01) were independent predictors of OS (data not shown). In addition, the only independent predictor of CSS was an FIF of ≥4 (HR: 3.64, 95% CI: 1.22-10.9, p = 0.02).

Treatment with phosphate binders (HR: 0.4104, 95% CI: 0.2362-0.713; p < 0.01) was the only independent predictor of nCFS based on the multivariate Cox proportional hazards regression analysis.

Multivariate analysis for cumulative incidence (including competing events) was performed using Fine-Gray proportional hazards regression analysis. An FIF of ≥4 (HR: 3.87, 95% CI: 1.43-10.45; p < 0.01) was an independent and significant factor in the multivariate analysis for the cumulative incidence of CSS (competing events to mortality without CSS).

An FIF of ≥4 (HR: 0.88, 95% CI: 0.48-1.62; p = 0.69) was not a significant factor in the multivariate analysis for the cumulative incidence of nCFS (competing events to all-cause mortality).

In the stepwise multiple regression analysis, an FIF of ≥4 was independently associated with the CRP level (correlation coefficient: 0.32; 95% CI: 0.11-0.53; p < 0.01), but not with the P, EPA, DHA, or ARA levels (data not shown).

## Discussion

This study obtained the following two findings: First, an FIF of ≥4 was significantly inferior to an FIF of ≤3 for CSS in HPs, and an FIF of ≥4 was independently associated with CSS.

Second, the incidence of new CVD onset during the 6-year observation period did not significantly differ between HPs with an FIF of ≥4 and those with an FIF of ≤3. Thus, an FIF of ≥4 might be one of the important risk factors for the development of CVD with high mortality in HPs.

The risk factors of CVD in HPs are a history of CVD, CKD mineral bone disorders, hyperphosphatemia, hyperlipidemia, smoking, hypertension, diabetes, alcohol consumption, chronic inflammation, and the presence of oxidants ^(1), (2), (3), (4), (5), (6), (7), (8), (9)^.

Similar to previous reports, our study showed that phosphate binder intake is an independent predictor of longer nCFS ^(1), (2), (3), (4), (5), (6), (7), (8), (9)^. Several CVD risk factors are associated with dietary habits, which have primary and secondary preventive effects against CVD ^(10)^. Moreover, appropriate dietary habits are effective in preventing CVD ^(10)^. However, it has been reported that dietary habits do not change significantly over time, as they are largely influenced by family preferences, food intake, income, and the person in charge of cooking ^(21), (22), (23), (24)^. On this basis, we investigated FIF only once. Therefore, dietary guidance should be given multiple times not only to HPs but also to their families regardless of age or sex.

Some reports show that a lower intake of fruits, vegetables, fish, and fiber and a higher intake of red meat, salt, and sugar, increases the risk of long-term hospitalization and mortality from CVD in HPs ^(4), (5)^. These reports have analyzed the association between dietary intake and dietary balance and the risk of mortality ^(4), (5)^. However, the results of these studies require complex calculations of dietary intake ^(1), (4), (5), (6), (8), (9)^, and they are challenging to apply in actual patient guidance.

In addition, EPA and DHA are CVD-preventive, and ARA is CVD-promotive ^(2), (3), (4), (6), (7), (8), (9)^. But deep-fried forms of the fish are not only associated with the benefits of preventing CVD ^(13), (25), (26)^, but also increased the risk of CVD. It has been reported that deep-frying fish increases both calorie intake and salt intake, and is associated with loss of the natural DHA and EPA in fish as they are replaced by cooking oil ^(26)^. Based on previous reports and the results of this study, it was inferred that a high FIF may be involved in dietary imbalance and increased ARA intake. Therefore, dietary guidance using only EPA, DHA, and ARA levels is challenging and limited because their content varies with food materials and cooking methods ^(2), (3), (4), (6), (7), (8), (9), (13), (25), (26)^.

In our study, there was no association between FIF ≥ 4 and P, EPA, DHA, or ARA levels, nor was there an association between nCFS or CSS and EPA, DHA, or ARA levels. The correlation coefficient between an FIF of ≥4 and CRP levels was 0.32, which indicated a weak correlation. Based on these findings and previous reports, it is impossible to explain the mechanism by which FIF ≥ 4 is one of the risk factors for the development of CVD with high mortality only by fatty acids and P in fish. However, the imbalance of intake with other foods due to an increase in fish intake may play a role in inducing inflammation. Thus, an FIF of ≥4 is one of the risks for developing CVD with high mortality and may apply to strict dietary guidance for HPs.

The current study had several limitations. This was a 6-year observational study using data from 148 HPs at a single institution with only 16 CVD mortalities and only on the FIF. Therefore, long-term observational studies should be conducted to investigate the frequency of the intake of foods such as meat, fish, vegetables, and fruits in a larger cohort of HPs.

We found that an FIF of ≥4 in HPs significantly worsened CSS and was independently associated with CSS. In addition, the incidence of a new CVD onset did not significantly differ between patients with an FIF of ≥4 and those with an FIF of ≤3 during the 6-year observational period. Therefore, an FIF of ≥4 could be one of the important risks for CVD with high mortality, and FIF may apply to strict dietary guidance for HPs.

## Article Information

### Conflicts of Interest

None

### Authors’ Contributions

Research conception and design: TA

Experiments: TA, TM, SF, SS, KM, DM, and YF

Data analysis: TA and SF

Interpretation of data: NY, RS, SA, MT, SS, HK, HF, MS, SH, TI, TS, TS, and HM

Writing the manuscript: TA

### Approval by Institutional Review Board (IRB)

This study was conducted following the Declaration of Helsinki and good clinician guidelines. Further, it was approved by the Ethics Committee of Nankai Medical Center (study number: NC-H27-001).

### Informed Consent

Consent for publication was obtained from all participants.

### Availability of Data and Materials

All data generated or analyzed during this study are included in this published article.
